# Novel Ligands for the Orphan Receptor GPR151 Modulate Morphine Action

**DOI:** 10.1111/gtc.70119

**Published:** 2026-05-08

**Authors:** Hijiri Yoshida, Takashi Suto, Hiroyuki Hirano, Hiroyuki Osada, Kazuto Nunomura, Bangzhong Lin, Shigeru Saito, Shigeki Takeda

**Affiliations:** ^1^ Division of Molecular Science, Graduate School of Science and Technology Gunma University Kiryu Gunma Japan; ^2^ Department of Anesthesiology Gunma University Graduate School of Medicine Maebashi Gunma Japan; ^3^ Chemical Resource Development Research Unit RIKEN Center for Sustainable Resource Science Saitama Japan; ^4^ Institute of Microbial Chemistry (BIKAKEN) Tokyo Japan; ^5^ Drug Innovation Center, Graduate School of Pharmaceutical Sciences The University of Osaka Suita, Osaka Japan

## Abstract

GPR151 is an orphan G‐protein‐coupled receptor expressed on specific neurons. Its expression has been reported to be upregulated in the dorsal root ganglia in mouse nerve injury models, suggesting a role in pain transmission. We used our unique ligand screening system to identify novel agonists and partial agonists for GPR151. The novel ligands, GUM3 and GUM4, have the psoralen ring and exhibited agonist activity (EC_50_ = 4.1 ± 1.0 μM) and highly weak partial agonist activity (EC_50_ = 0.81 ± 1.46 μM), respectively, in the [^35^S]‐GTPγS binding assay. Both ligands induced GPR151 internalization and increased reporter gene expression in Chinese hamster ovary cells transiently expressing human GPR151 upon ligand stimulation. Activity evaluation employing structural analogs provided insights into a series of ligand structure–activity relationships. Administration of the unique ligands had no effect on thermal hyperalgesia in rats; however, an agonist and a partial agonist enhanced and attenuated the analgesic effect of morphine, respectively, when co‐administered. These findings not only reveal the physiological role of GPR151 in pain transmission but also demonstrate its potential as a novel drug target for pain control. Our findings indicate that the development of GPR151 ligands can contribute to the reduction of opioid use.

## Graphical abstract

The new GPR151 ligands altered the analgesic effects of morphine.

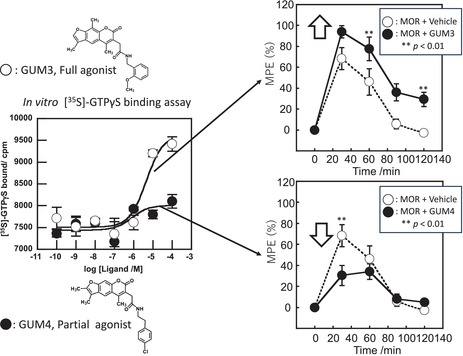

## 1 Introduction

G‐protein‐coupled receptors (GPCRs) constitute one of the largest protein super‐families that specifically recognize various types of stimuli and chemicals. GPCRs transmit signals to cells via G proteins and induce various changes in cells. GPCRs are major drug targets because they control cellular states with their ligands. To explore new drug targets and develop drug candidate compounds, we identified GPCR genes in the human genome (Takeda et al. 2002) and developed a unique ligand screening system (Takeda et al. 2004). In our ligand screening system, we express fusion proteins of GPCR and G protein α‐subunit using insect cells and measure the agonist‐dependent [^35^S]‐GTPγS binding activity of membrane fractions expressing the fusion proteins. This system is simple and relatively inexpensive to identify new ligand compounds. Because the system is in vitro without requiring living cells for the assay, the activity can be measured even for synthetic chemical compounds that have low water solubility and are dissolved in organic solvents, such as dimethyl sulfoxide (DMSO). In addition, the constitutive activity of the fusion protein can be used to identify inverse agonists in the absence of agonists (Yanai et al. 2016; Sakai et al. 2022). These features are, particularly advantageous for screening new ligand candidates during early stages of drug development. We have used this unique system to develop novel ligands for various GPCRs. In a previous study, we developed novel opioid receptor agonists, designated GUM1, and validated their analgesic effects in rats (Nikaido, Kurosawa, et al. 2015). A small‐molecule compound similar to the novel ligand identified in that study was marketed as Oliceridine in 2020 by Trevena (Liu et al. 2025). A novel agonist of bile acid receptor (GPBA), GUM2, was also developed (Enomoto et al. 2017). These findings demonstrate the utility of our ligand screening system for drug discovery.

All human GPCRs were identified by comprehensive identification after the Human Genome Project was completed (Takeda et al. 2002). However, various GPCRs have no endogenous ligands identified, and details of their physiological actions are unknown, thus being referred to as orphan GPCRs. As few ligands for orphan GPCRs have been identified, their study has been limited to genetic analyses such as single nucleotide polymorphism analysis, organ‐specific expression analysis, and genetically modified animals. Therefore, ligands specific for orphan GPCRs must be discovered to further clarify their physiological functions. Super‐conserved receptors expressed in brain (SREB) are an orphan GPCR subfamily that is highly expressed in the brain. We previously reported novel synthetic ligands for three orphan GPCRs, namely, GPR27, GPR85, and GPR173, which belong to the SREB subfamily (Yanai et al. 2016; Sakai et al. 2022) and have been related to analgesic effects (Xu et al. 2024).

GPR151 is another orphan GPCR that, like SREB compounds, is highly expressed in the brain and has been reported to participate in pain transmission. GPR151 was reported to be specifically expressed in the habenula region of the nervous system (Broms et al. 2015). In mouse neuropathic pain models, GPR151 mRNA expression was strongly upregulated in the dorsal root ganglia (Holmes et al. 2017). Analysis of GPR151 knockout mice also showed that GPR151 in the dorsal root ganglia was important for neuropathic pain processing (Xia et al. 2021). In intracellular signaling, GPR151 couples with the Gαi protein involved in pain and mediates the downstream mitogen‐activated protein kinase (MAPK) pathway (Jiang et al. 2021). Subsequently, GPR151 induces inward Ca^2+^ currents through P2X3 ion channels, which are involved in neuropathic pain by increasing neuronal excitability (Xia et al. 2021). Therefore, several studies have been focused on the relationship between GPR151 and pain (Jiang et al. 2018; Xu et al. 2024), and GPR151 was of great interest to those of us who have been involved in the development of novel synthetic opioids (Nikaido, Kurosawa, et al. 2015). However, understanding the functional mechanisms of GPR151 in pain is still incomplete. As no ligand compounds specific to GPR151 have been identified, its drug development potential has remained unclear. We believe that a breakthrough in understanding the physiological function of GPR151 requires discovering specific ligands for GPR151.

In this paper, we report the development of novel ligands for GPR151. Hit compounds identified in vitro systems often fail to exhibit activity in vivo. However, GUM3 and GUM4, identified as novel GPR151 ligands using our proprietary in vitro GPCR ligand screening method, demonstrated pharmacological activity in in vivo measurements using rats. These results demonstrate that our novel ligands for GPR151 are useful for studying the physiological functions of GPR151 and that GPR151 is a valuable new drug target. Our novel synthetic GPR151 agonist enhances the antinociceptive effects of morphine, while the weak partial agonist attenuates the effect of morphine. These findings suggest that GPR151 is involved in acute pain transmission and that specific ligands for GPR151 can contribute to reducing opioid use for pain management. Drugs that modulate morphine action are expected to prevent serious opioid side effects, such as respiratory depression, and mitigate the development of opioid tolerance. We expect that this study will contribute to the development of new analgesic management agents targeting GPR151.

## 2 Results

### 2.1 [ ^35^S]‐GTPγS Binding Assay for Novel Ligand Screening

**FIGURE 1:**
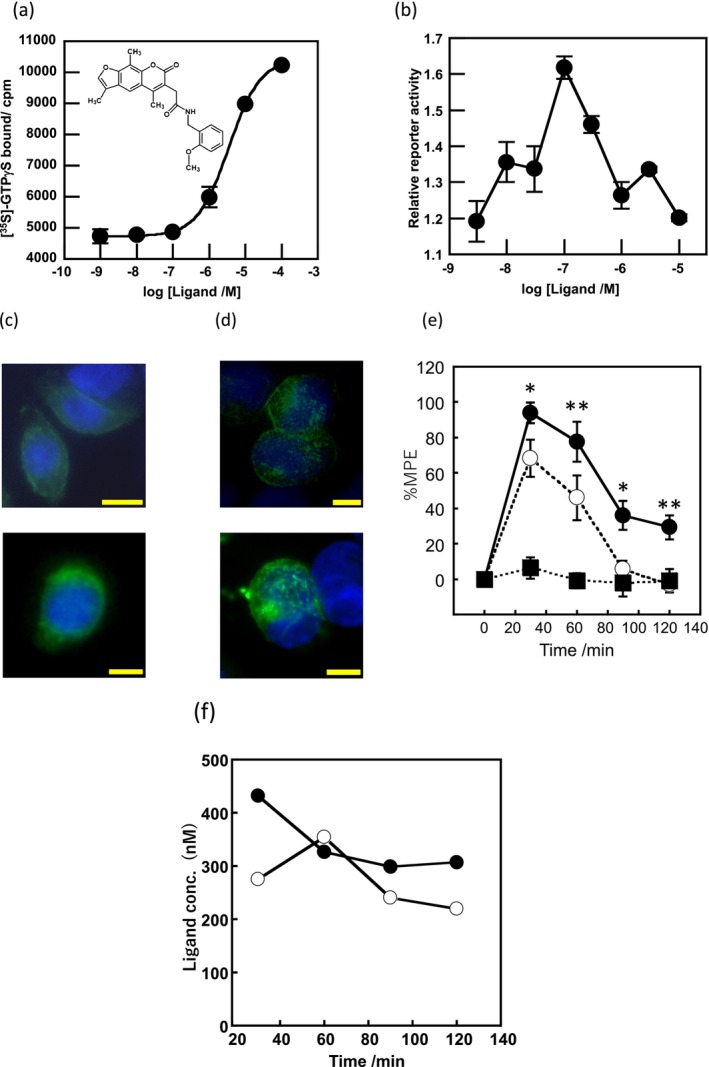
In vitro and in vivo characterization of GUM3. (a) Stimulation of [^35^S]‐GTPγS binding in the presence of GUM3 in membrane fractions expressing GPR151‐Giα. Datapoints represent the mean ± standard error of the mean across three measurements and are representative of at least three independent experiments. The chemical structure of GUM3 is displayed in the box. (b) Reporter gene assay using transiently transfected CHO cells with an HA‐tagged GPR151 expression vector. Each datapoint represents mean ± standard error of the mean across three measurements, representative of at least three independent experiments. Representative images of GPR151 localization by fluorescence microscopy in transiently transfected CHO cells with HA‐tagged GPR151 expression vector treated for 6 h with (c) vehicle and (d) 100 μM GUM3, showing GPR151 internalization after GUM3 stimulation. The scale bars correspond to 10 μm. (e) Effects of GUM3 on thermal pain in native rats. Rats were administered morphine (closed squares, *N* = 8), GUM3 (open circles, *N* = 8), or both morphine and GUM3 (closed circles, *N* = 7), and PWL to heat stimulation was measured. Data are expressed as mean ± standard error of the mean. Individual means for morphine alone versus morphine and GUM3 pairs were compared two‐way repeated‐measures ANOVA, followed by multiple comparisons. The *p*‐value was corrected versus morphine alone using Bonferroni's method and was 0.0454, 0.0091, 0.0132, 0.0064 at 30, 60, 90, and 120 min, respectively. *p < 0.05, **p < 0.01. (f) Time course of serum GUM3 concentrations. White circles and black circles represent individual rats, respectively. GUM3 was persistently present in serum during the antinociception test. Drug concentrations during antinociception test correlated closely with the active GUM3 concentrations observed in the CHO cell assay.

Ligand screening for GPR151 was performed using the RIKEN pilot library (Kato et al. 2012), which contains 400 compounds (Nikaido, Kurosawa, et al. 2015; Yanai et al. 2016; Enomoto et al. 2017) (https://www.npd.riken.jp/crdu/en/c‐distribution.html). The ligand‐dependent increase in [^35^S]‐GTPγS binding to cell membrane fractions containing the GPR151‐Giα fusion protein was measured in duplicate in the presence of approximately 0.1 mg/mL of each compound from the library. The results showed that two hit compounds exhibited values more than twice the standard deviation above the mean radio counts. The same screening was also performed for three additional fusion proteins: human GPBA‐Gsα (Enomoto et al. 2017), human μ‐opioid receptor‐Giα (Nikaido, Kurosawa, et al. 2015), and human GPR84‐Giα (middle chain free fatty acid receptor) (Nikaido, Koyama, et al. 2015). However, no compound exhibited activity against these fusion proteins. These results suggested that the two hit compounds were not nonspecific ligands for GPCRs or Gα proteins. We also conducted dose‐dependent measurements to determine the EC_50_ values of these two small‐molecule hit compounds. NPD12440, named GUM3, showed an EC_50_ of 4.1 ± 1.0 μM (Figure 1a). NPD13617, which has a similar chemical scaffold to GUM3, showed an EC_50_ of 25 ± 1.3 μM (Figure S1).

### 2.2 Reporter Gene Assays and Fluorescence Study

To validate that GUM3 is active and functional against GPR151 in living cells, Chinese hamster ovary (CHO) cells transiently expressing GPR151 were stimulated with GUM3. The activity was assessed using MAPK/ERK and serum response element‐mediated intracellular signaling pathways as indicators of reporter gene expression (Mashiko et al. 2019). In CHO cells transfected with a mock vector (pcDNA3.1), no reporter gene expression was induced at any GUM3 concentration. In contrast, GUM3 induced an increase in reporter gene expression at concentrations ranging from 10 to 300 nM (Figure 1b). GUM3 activity was not observed in the [^35^S]‐GTPγS assay at 100 nM, but it was detected in the CHO reporter gene assay. These results suggested that CHO cells were activated at more than 10‐fold lower ligand concentrations compared with those used in the [^35^S]‐GTPγS binding assay. Moreover, no activity was observed at 10 μM, suggesting that cell activation was only within a narrow concentration range. We concluded that signal amplification through intracellular signaling occurred and that GUM3 likely induces strong desensitization.

GPCRs are typically desensitized by agonist‐induced activation. During activation, they are translocated to the endoplasmic reticulum within the cell. As we did not observe activation of GPR151 by GUM3 in CHO cells at a concentration of 10 μM, we aimed to confirm whether this result was due to desensitization caused by receptor internalization. CHO cells expressing GPR151 with an HA‐tagged extracellular N‐terminus were treated with 100 μM GUM3 or vehicle and stained with a fluorescent antibody after 6 h, matching the time point used in the reporter gene assay. We observed that GPR151 was localized to the cell membrane in the vehicle‐treated group (Figure 1c), whereas GUM3 induced migration of GPR151 into the cytoplasm under a fluorescence microscope (Figure 1d). These findings confirmed that GUM3 induced intracellular translocation of GPR151.

### 2.3 Effect of GUM3 on Acute Antinociceptive Effects of Morphine

The effects of GUM3 on acute thermal pain were studied in vivo using naïve rats. Results of two‐way repeated‐measures analyses of variance (ANOVAs) were summarized in Table 1. A single intraperitoneal injection of GUM3 (1 mg/kg) did not alter the paw withdrawal latency (PWL) of rats following radiant heat stimulation. Next, morphine (10 mg/kg) and GUM3 (1 mg/kg) were co‐administered to investigate the effect of GUM3 on the analgesic action of morphine. While GUM3 alone had no effect on PWL, coadministration of morphine and GUM3 resulted in a statistically significant prolongation of PWL compared to morphine alone at 60–120 min after drug administration. *p*‐value was corrected versus morphine alone using Bonferroni's method and was 0.0454, 0.0091, 0.0132, 0.0064 at 30 min, 60, 90 and 120 min, respectively (Figure 1e). Put differently, GUM3 potentiated the analgesic effects of morphine. The serum GUM3 concentration was measured using liquid chromatography coupled with tandem mass spectrometry (LC–MS/MS) every 30 min after drug administration (Figure 1f). These results showed that the serum GUM3 concentration at the time of analgesic enhancement closely matched the active GUM3 concentration observed in the CHO cell assay (Figure 1b).

**TABLE 1:** Two‐way repeated‐measures ANOVA results for the antinociception test following drug administration.

Drug	Source of variation	*F*	*p*
Morphine + vehicle vs Morphine + GUM3 vs Saline + GUM3	Group	*F* (2, 20) = 21.36	< 0.0001
	Time	*F* (4, 80) = 51.57	< 0.0001
	Interaction	*F* (8, 80) = 12.15	< 0.0001
Morphine + vehicle vs Morphine + GUM4 vs Saline + GUM4	Group	*F* (2, 20) = 7.10	0.0047
	Time	*F* (4, 80) = 24.11	< 0.0001
	Interaction	*F* (8, 80) = 8.02	< 0.0001

### 2.4 Identification of Potent Ligands Based on GUM3 Derivatives

The confirmation GUM3's agonist activity in CHO cells and its ability to enhance the analgesic effects of morphine in rats highlighted the pharmacological importance of GPR151 and GUM3. Therefore, we explored additional GPR151 ligands with distinct properties among compounds structurally similar to GUM3. Unfortunately, our compound library contained very few structural analogs of NPD13617. Furthermore, NPD13617 exhibited lower affinity than GUM3, so subsequent evaluations using structural analogs primarily focused on GUM3. Structural homologs of GUM3 were first identified from the RIKEN compound library and classified into 80 structural clusters based on their molecular features. Two representative compounds were then selected from each cluster to construct a focused library of 160 compounds. We measured the increased uptake of [^35^S]‐GTPγS for GPR151‐Giα at a final concentration of approximately 10 μM for the 160 selected compounds and expressed the results as the relative activity of each compound, with the activity of GUM3 set at 100% (Table 2). The results provided insights into structure–activity relationships (see Section 3). Compounds preferentially excluded from the ligand candidates included 29 compounds bearing large substituents, such as phenyl or tert‐butyl groups at the third carbon from the furan ring, which significantly reduced activity. Similarly, 46 compounds in which the furan ring structure was disrupted and replaced with a bicyclic ring completely lost agonist activity. Conversely, we identified 12 compounds with activity comparable to GUM3, exhibiting EC_50_ values of approximately 2–8 μM. As these 12 compounds were in clusters 54, 55, 56, and 58, the remaining 42 compounds in these clusters were also assessed using the [^35^S]‐GTPγS binding assay. Among these 42 compounds, six showed efficacy similar to GUM3, with comparable or higher affinity. In particular, NPD4841 showed the lowest EC_50_ of 0.28 ± 0.09 μM (Table 3). This compound shared a similar chemical scaffold with GUM3 and demonstrated the highest affinity for GPR151, approximately 50‐fold lower than that of GUM3 (Figure S1). Interestingly, the EC_50_ value for NPD12827, which possessed a chemical scaffold similar to GUM3 but with two fewer methyl groups than NPD4841, was 2.0 ± 0.15 μM.

**TABLE 2:** Summary of the activity of structural analogs with (a) two methyl and (b) three methyl groups on the psoralen ring.

	Name	Structure (R)	Relative activity^a^
*(a) Two methyl groups*			
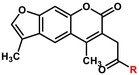	NPD12680	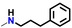	112%
	NPD12827	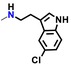	99%
	NPD4524	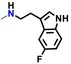	76%
	NPD12850	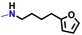	52%
	NPD3832	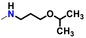	40%
	NPD12931	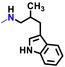	39%
	NPD12475	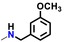	34%
	NPD4636	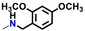	23%
	NPD12508	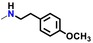	31%
	NPD9943	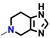	26%
	NPD2996	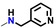	20%
	NPD12798	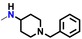	19%
	NPD5007	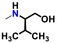	14%
	NPD2680	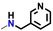	13%
	NPD7218	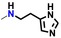	13%
	NPD12858	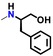	12%
	NPD3760	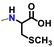	9%
*(b) Three methyl groups*			
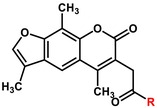	NPD12803	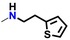	105%
	NPD4256	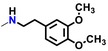	97%
	NPD12440 (GUM3)	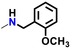	100%
	NPD3946	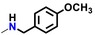	82%
	NPD3246	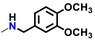	73%
	NPD2871	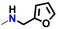	82%
	NPD4428	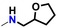	53%
	NPD2463	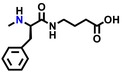	19%
	NPD13443	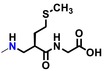	17%
	NPD13629	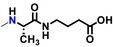	16%
	NPD4220	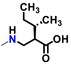	13%
	NPD2926	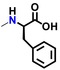	12%
	NPD12854	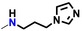	11%
	NPD12141	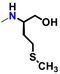	5%
	NPD3726	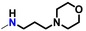	0%

^a^ Relative activity is expressed as the amount of [^35^S]‐GTPγS uptake at a concentration of 100 μM per compound, with GUM3 set as 100%.

**TABLE 3:** Six compounds exhibiting similar activity to GUM3 among 42 compounds belonging to clusters 54, 55, 56, and 58.

Name	Structure	EC_50_
NPD3946	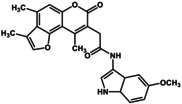	2.0 ± 0.57 μM
NPD4386	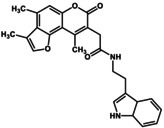	1.5 ± 0.040 μM
NPD4886	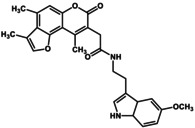	1.9 ± 0.45 μM
NPD3915	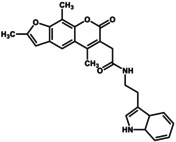	0.54 ± 0.42 μM
NPD12847	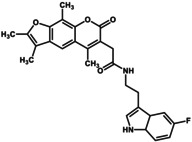	0.71 ± 0.29 μM
NPD4841	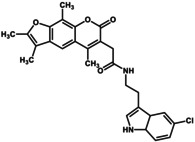	0.28 ± 0.09 μM

### 2.5 In Vitro and In Vivo Characterization of GUM4

**FIGURE 2:**
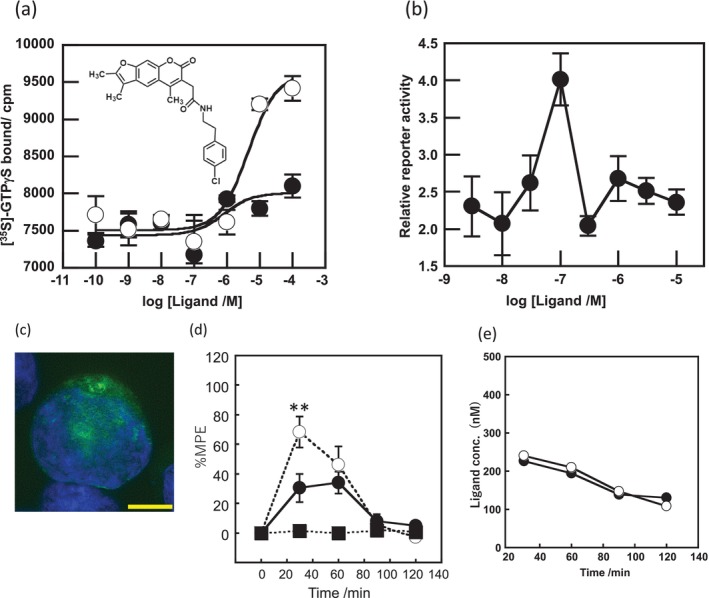
Evaluation of GUM4, as described in Figure 1. (a) [^35^S]‐GTPγS binding assay. The chemical structure of GUM4 is displayed in the box. (b) Reporter gene assay. (c) GUM4‐stimulated intracellular translocation. The scale bar corresponds to 10 μm. (d) Effects on thermal pain in native rats. Rats were administered morphine (closed squares, *N* = 8), GUM4 (open circles, *N* = 6), or a combination of morphine and GUM4 (closed circles, *N* = 9), and PWL to heat stimulation was measured. Data are expressed as mean ± standard error of the mean. Individual means for morphine alone versus morphine and GUM4 pairs were compared two‐way repeated‐measures ANOVA, followed by multiple comparisons. The *p*‐value was corrected versus morphine alone using Bonferroni's method and was less than 0.0001 at 30 min. **p < 0.01. (e) Time course of serum GUM4 concentrations. White circles and black circles represent individual rats, respectively. GUM4 was persistently present in serum during the antinociception test. Drug concentrations during antinociception test correlated closely with the active GUM4 concentrations observed in the CHO cell assay.

To elucidate the physiological function of GPR151, we considered it necessary to identify not only agonists such as GUM3 and NPD4841, but also a variety of ligands. We have previously reported examples in which inverse agonists were readily identified without any agonists using a screening method involving fusion proteins of GPCRs and G proteins (Yanai et al. 2016; Sakai et al. 2022). However, during the analysis of GUM3 structural analogs, no candidates for inverse agonists of GPR151 were identified. On the other hand, potential compounds acting as partial agonists for GPR151 were found among GUM3 structural analogs. Unfortunately, we were unable to obtain sufficient quantities of these compounds for further analysis. Therefore, we sought commercially available GUM3 structural analogs, and serendipitously discovered that GUM4 exhibits extremely weak partial agonist activity. To determine the EC_50_ value of GUM4, a dose‐dependent [^35^S]‐GTPγS binding assay was performed. GUM4 exhibited highly weak partial agonist activity. Owing to the low efficacy and the small difference in radio counts between pre‐ and post‐activation, the EC_50_ measurement was slightly less precise, resulting in a value of 0.81 ± 1.5 μM (Figure 2a). Despite this reduced accuracy, the potency of GUM4 was similar to that of NPD12827 and NPD4841. Reporter gene assays using CHO cells expressing GPR151 demonstrated that GUM4 induced an increase in reporter gene expression at 100 nM (Figure 2b). Thus, CHO cells were activated by GUM4 at ligand concentrations approximately eightfold lower than the EC_50_ value obtained from the [^35^S]‐GTPγS binding assay. Additionally, fluorescence microscopy revealed internalization of GPR151 similar to that observed with GUM3, confirming that GUM4 exhibits weak but significant agonist activity (Figure 2c). The effects of GUM4 on acute thermal pain were studied in vivo using naïve rats. Results of two‐way repeated‐measures ANOVA were summarized in Table 1. Similar to GUM3, GUM4 alone did not change pain‐related behavior. However, when morphine and GUM4 were coadministered, the PWL at 30 min postadministration was significantly shorter. *p*‐Value was corrected versus morphine alone using Bonferroni's method and was less than 0.0001 at 30 min (Figure 2d). In other words, GUM4 counteracted the analgesic effects of morphine. As well as GUM3, the serum GUM4 concentration was measured using liquid chromatography coupled with LC–MS/MS every 30 min after drug administration (Figure 2e). These results demonstrated that serum GUM4 concentrations during analgesic potentiation were closely correlated with the active GUM4 concentrations observed in the CHO cell assay (Figure 2b). Although GUM4 demonstrated highly weak partial agonist properties in vitro, in vivo results showed that it produced pharmacological effects opposite to those of GUM3 (i.e., antagonist‐like effects). It is known that weak partial agonists can exhibit antagonist‐like activity in animal models, which occurs because when receptors are occupied by weak partial agonists, they fail to elicit their native activity. For example, partial agonists with in vivo antagonistic effects have been reported for dopamine receptor ligands (Meyer 2024). Pentazocine acts as a partial agonist at the μ‐receptor and thus counteracts morphine's effects (Li et al. 2023). However, the observed phenomenon in which GUM4 attenuates the analgesic effects of morphine is more complex to understand because it involves different receptor systems, such as GPR151 and the opioid receptors.

## 3 Discussion

In an earlier study, we successfully identified novel ligand compounds for GPCRs by expressing GPCR‐Gα fusion proteins in insect cells and using them in [^35^S]‐GTPγS binding assays, which served as the screening system in the present study (Takeda et al. 2004). Additionally, we applied a novel clustering analysis to refine our ligand development strategy. In this analysis, we narrowed the range of compounds by categorizing those with similar chemical structures and then assessed the selected representative to identify promising clusters. This ligand improvement method, aimed at obtaining optimized chemical structures, successfully proved efficient and led to the identification of NPD4841, which exhibited up to a 50‐fold increase in potency compared with the initial hit, GUM3. The activity evaluation of an extracted library comprising 160 compounds, selected as structural analogs of GUM3 through cluster analysis, provided insights into a series of ligand structure–activity relationships. Compounds demonstrating full agonist activity for GPR151 were mainly composed of a few types of psoralen rings. Among the 160 GUM3 structural analogs, 15 and 16 compounds were classified as containing three and two methyl groups in the psoralen ring, respectively. The side chain structure, linker length, and relative activity of each compound compared to GUM3 are summarized in Table 2, with GUM3's activity considered as 100%. For compounds with two methyl groups in the psoralen ring to exhibit sufficient agonist activity, the linker from the amide group to the benzene ring at the end of the side chain consisted of three carbons (Table 2). In contrast, for compounds with three methyl groups in the psoralen ring, the linker from the amide group to the aromatic ring—essential for activity—comprised one or two carbons (Table 2). In both cases, the substituent attached to the benzene ring influenced compound activity; however, a wide variety of substituents appeared to be allowed (Figure S2). Notably, the main skeleton of our ligands is the same as that of the natural product psoralen, which is present in foods such as celery, parsley, figs, and citrus fruits.

We confirmed that GUM3 and GUM4 are not ligands for the μ‐opioid receptor, the κ‐opioid receptor, or the δ‐opioid receptor by measuring their [^35^S]‐GTPγS binding activity (Figure S3). These results indicated that the modulation of morphine effects demonstrated by our novel ligands was not mediated by opioid receptor activity. We also examined whether GPR151 directly interacts with the opioid receptors. As an example of opioid receptor activity modulation by other GPCRs, the orphan receptor GPR88 has been reported to form heterodimers with the μ‐opioid receptor (Laboute et al. 2020). We suspected that the opioid receptors might form heterodimers with GPR151, with their activity potentially regulated by GUM3 and GUM4. To investigate this possibility, we performed [^35^S]‐GTPγS binding assays using cell membranes co‐expressing one of the opioid receptors and GPR151. We found that GUM4 was a very weak partial agonist, closer to an antagonist. Therefore, if the opioid receptors and GPR151 form a heterodimer, we expected that GUM4 binding to GPR151 would inhibit morphine‐induced opioid receptor activations. As results, GUM4 did not inhibit opioid receptor activations (Figure S4). Therefore, the effects of GPR151 ligands on pain signaling in the opioid receptors were considered distinct from those mediated by heterodimer formation between GPR88 and the opioid receptors. Meanwhile, elucidating the mechanism by which GUM4 attenuates morphine effects remains complex. In another preliminary experiment, no statistically significant results were obtained indicating that GUM3 or GUM4 act as allosteric modulators of the opioid receptors. Figure S4 also showed that on cell membranes co‐expressing GPR151 and the δ‐receptor, morphine‐induced δ‐receptor activation tended to be slightly enhanced (rather than reduced) in the presence of GUM4. This phenomenon is difficult to discuss at present and may require further investigation. While many reports generally state that “activation of the δ‐receptor enhances analgesic effects,” it should be noted that the δ‐receptor is expressed on peripheral nociceptive C‐fibers and dorsal root ganglia, and that there are reports suggesting the δ‐receptor may regulate pain suppression through modulation of GABA release and control of neural excitability (Alexander and Bender 2025). The objective of this paper is to demonstrate that the compounds identified through in vitro screening also exhibit pharmacological activity in vivo. Elucidating the detailed mechanisms underlying this activity will serve as the next step toward understanding the physiological role of GPR151.

As no endogenous ligands for GPR151 have been identified, research on this receptor has been largely focused on gene analyses. The localized expression of GPR151 in the habenula has attracted particular interest, and study using knockout mice have revealed its involvement in promoting social reward (Allain et al. 2022) and drug dependence (Antolin‐Fontes et al. 2020). GPR151 mRNA is highly expressed in brain regions that play key roles in pain, stress, memory, and depression (Ignatov et al. 2004; Quina et al. 2009; Hikosaka 2010). Previous studies have also reported that nerve injury increases GPR151 mRNA expression (Holmes et al. 2017; Xu et al. 2024). Hence, GPR151 signaling is likely involved in pain processing and the development of nerve injury–related pain. More interestingly, GPR151 is coupled with P2X3 ion channels in IB4‐positive neurons, which constitute one of two broad classes of C‐fiber sensory neurons involved in pain transmission (Xia et al. 2021). Suzetrigine (VX‐548), a selective inhibitor of the NaV1.8 sodium channel, is involved in pain transmission pathways in the peripheral nervous system as an analgesic without the risk of dependency (Hang Kong et al. 2024; Vaelli et al. 2024). Similar to the antipain mechanism of Suzetrigine, we suppose that GPR151 contributes to pain modulation by regulating ion channels. We measured concentrations of the two novel ligands not only in serum but also in cerebrospinal fluid (CSF). For highly hydrophobic small molecules, CSF drug concentrations do not exactly match brain concentrations but show reasonable correlation. Therefore, we estimated the concentration of the compound that crossed the blood–brain barrier into the brain based on its concentration in CSF. At 30 and 120 min postadministration, CSF was collected from two individual rats in addition to blood, and the concentrations of GUM3 and GUM4 contained therein were measured. The results showed that only trace amounts of GUM3 and GUM4 were detected in the CSF. This led us to conclude that despite their high hydrophobicity, our novel ligands did not cross into the brain. These facts also align with our consideration that our compounds act on GPR151 expressed in peripheral tissues, such as the C‐fiber sensory neurons and dorsal root ganglia, to modulate pain signals (Xia et al. 2021). We surmised that GUM3 and GUM4 exerted their pharmacological effects by acting on peripheral GPR151, but this is merely one possible explanation for our results.

No significant differences have been reported between wild‐type and knockout mice in acute‐pain‐like behavior (Holmes et al. 2017). However, the effects of morphine on heat‐induced acute nociception were affected in rats treated with a synthetic small molecule ligand for GPR151, namely GUM3 and GUM4. In other words, our findings indicate that GPR151 ligands modulated the action of morphine in an acute pain model. Not only acute‐pain, but also chronic pain is a serious medical problem (Scholz and Woolf 2002). The analgesic efficacy of morphine diminishes and becomes limited as chronic pain treatment progresses (Kimura et al. 2014; Hiroki et al. 2022). The development of GPR151 agonists, which increase the efficacy of morphine, may enable the continued use of opioids for chronic pain management. Based on reports indicating that nerve injury increases GPR151 expression (Holmes et al. 2017), we initially hypothesized that administration of the novel agonist GUM3 would decrease the analgesic effects of morphine. Conversely, in vivo experiments showed that GUM3 potentiated the analgesic effects of morphine. One possibility for these discrepancies is that GPR151 may be differentially involved in pain transmission in chronic pain models of nerve injury animals and in acute pain models induced by thermal stimulation. The discrepancies may also be related to the species differences between mice and rats in the expression response of GPR151 to nerve injury (Xu et al. 2022), while the *GPR151* gene is evolutionarily conserved. We believe that a thorough investigation of GPR151's role in pain transmission is essential to elucidate mechanisms underlying our observations. Our experimental findings indicated that it was highly likely that the pharmacological effects of GUM3 and GUM4 were mediated via GPR151. Further verification is required to support this view. For example, studies involving nerve‐specific expression of GPR151 via viral gene transfer in GPR151 knockout animals, followed by administration of GPR151 ligands, could provide valuable insights. Comparing animal study results using GUM3 (EC_50_ = 4.1 μM) and NPD4841 (EC_50_ = 0.8 μM) is pharmacologically significant. Understanding the mechanism by which GPR151 full agonists enhance morphine's analgesic effects while partial agonists exhibit antagonistic‐like effects is a key focus of our ongoing research, which will be the subject of our next paper. Opioid abuse remains a significant social problem in several countries. Additionally, although opioids are clinically excellent analgesics, they can cause serious side effects, such as respiratory depression, in certain cases. If GPR151 ligands can be administered to reduce opioid dosage while maintaining analgesic efficacy, they may help address several of the current challenges associated with opioid use. GUM3 and GUM4 will be valuable for further investigating the physiological functions of GPR151 and exploring its potential in drug development to improve the treatment of various diseases. We believe that these ligands and our findings highlight the importance of GPR151 as a promising target for drug discovery.

During antinociception measurements, biting and pulling at the forelimb nails was frequently observed in GUM3‐treated rats. This behavior was not observed in the vehicle‐treated group, indicating that this phenomenon resulted from the pharmacological action of GUM3. We considered that these observations may be related to a potential association between GPR151 and anxiety or stress, as well as the ability of GUM3 to potentiate anxiety and stress in animals. Further behavioral studies are necessary to better understand the possible side effects of GUM3 administration. We currently propose that GUM3 and GUM4 act on GPR151 expressed on peripheral C‐fibers and dorsal root ganglia to modulate the effects of morphine. Given that GPR151 exhibits important physiological actions not only centrally but also peripherally, it represents an excellent drug discovery target (DePasquale et al. 2025). Furthermore, its critical role in the central nervous system makes the design and development of novel ligands with high water solubility and brain permeability a key focus in drug discovery. Therefore, our next goal is to develop improved ligands with enhanced water solubility based on high‐affinity compounds such as NPD4841, and to conduct pharmacological studies, pharmacokinetic analyses, and behavioral experiments using these ligands.

Moreover, identifying endogenous ligands for GPR151 will be critical for understanding its physiological functions. We have previously reported that GPR151 is activated under low pH conditions in vitro (Mashiko et al. 2019), but the possible contribution of this phenomenon to its physiological role remains to be verified. All the facts presented above suggest that GPR151 represents a potential target for drug discovery. Several studies on orphan GPCRs, including GPR151, will be essential for advancing biology and pharmacological understanding. All the facts presented above suggest that GPR151 represents a potential target for drug discovery. Further studies on orphan GPCRs, including GPR151, will be essential for advancing biology and pharmacological understanding.

## 4 Experimental Procedures

### 4.1 Chemicals

The pilot library containing 400 chemicals for the initial ligand screening and 160 structural analogs of the hit compounds was retrieved from a ligand database maintained by the RIKEN Natural Products Bank (NPDepo). The agonist GUM3 (*N*‐[(2‐methoxyphenyl)methyl]‐2‐{3,5,9‐trimethyl‐7‐oxo‐7H‐furo[3,2‐g]chromen‐6‐yl}‐acetamide; catalog number STL462626 was procured from Vitas‐M Laboratory (Hong Kong). The partial agonist GUM4 (*N*‐[2‐(4‐chlorophenyl)ethyl]‐2‐{2,3,5‐trimethyl‐7‐oxo‐7H‐furo[3,2‐g]chromen‐6‐yl}cacetamide); catalog number 1099911) was procured from OTAVA chemicals (Lithuania).

### 4.2 Preparation of Cell Membranes Containing GPR151‐Giα Fusion Protein

GPR151‐Giα fusion proteins were synthesized using the baculovirus‐Sf9 expression system as previously reported (Takeda et al. 2003, 2004). The open reading frame of the human *GPR151* gene was fused to bovine Gi1α cDNA by a two‐step polymerase chain reaction using KOD polymerase (Toyobo, Japan). The fusion gene was cloned between the NotI and XbaI multiple cloning sites of pFASTBacI (Invitrogen, USA). All DNA constructs were confirmed by DNA sequencing. These plasmids were then used to prepare Bacmids containing the GPR151‐Giα fusion gene with the Bac‐to‐Bac kit (Invitrogen). The resulting Bacmid was transfected into Sf9 cells using Cellfectin (Invitrogen) to generate recombinant baculovirus carrying the GPR151‐Giα fusion gene. The virus was amplified by infecting Sf9 cells cultured at 28°C in CELL 420 medium (Sigma‐Aldrich) supplemented with 5% fetal bovine serum (GE Healthcare). The amplified virus culture medium was added to floating cultures of Sf9 cells at approximately 1:100 dilution and allowed to infect the cells. After 96 h of incubation, infected cells were collected and used for preparation of the cell membrane fraction. Sf9 cells expressing the GPR151‐Giα fusion protein were resuspended in a membrane preparation buffer (50 mM HEPES‐KOH with pH 7.0, 10 mM MgCl_2,_ 1 mM phenylmethylsulfonyl fluoride, 10 μg/mL leupeptin, 10 μg/mL pepstatin, 1 mM ethylenediaminetetraacetic acid) and homogenized on ice. The ruptured cell suspensions were centrifuged at 100,000 rpm for 90 min (CS120FNX ultracentrifuge, Hitachi). The resulting pellet was resuspended in the same membrane preparation buffer and stored at −80°C as a membrane fraction expressing the GPR151‐Giα fusion protein. Cell membranes expressing the μ‐opioid receptor‐Giα, the κ‐opioid receptor‐Giα, and the δ‐opioid receptor‐Giα were similarly prepared (Nikaido, Kurosawa, et al. 2015). Protein concentrations were determined using a protein assay kit (Bio‐Rad).

### 4.3 Guanosine 5′‐*O*‐(3‐Thiotriphosphate) ([ ^35^S]‐GTPγS) Binding Assay

To measure the ligand‐dependent enhancement of [^35^S]‐GTPγS binding, membranes expressing the GPR151‐Giα fusion protein (equivalent to 20 μg of total protein) were added to 96‐well microplates with a total volume of 100 μL per well. The reaction mixture contained 20 mM 4‐(2‐hydroxyethyl)‐1‐piperazineethanesulfonic acid potassium hydroxide buffer (HEPES‐KOH) (pH 8.0), 1 mM ethylenediaminetetraacetic acid, 160 mM NaCl, 1 mM dithiothreitol [^35^S]‐GTPγS (1250 Ci/mmol; PerkinElmer), 0.1 μM guanosine diphosphate, 10 mM MgCl_2_, and appropriate concentrations of the test compounds dissolved in DMSO. The plates were incubated at 30°C for 30 min. The final concentration of each compound used for screening was 100 μM. The [^35^S]‐GTPγS bound to the membrane fraction was captured on a GF/B glass fiber filter (PerkinElmer) and analyzed using a liquid scintillation counter (TopCount, PerkinElmer). Measurements were performed in triplicate at each ligand concentration, and the mean and standard deviation were computed. The resulting datapoints were fitted to an EC_50_ curve using Equation (1), where *X* is the ligand concentration, *Y* is the average observed radio counts, *A* is the calculated apparent resting state, and *A* + *B* is the calculated radio count of the activated state. Curve fitting was calculated using KaleidaGraph (Synergy Software).

(1) $$Y=A+\frac{B}{1+\left(\frac{\mathrm{EC}50}{X}\right)}$$

### 4.4 Reporter Gene Assays

The open reading frame of the human GPR151 gene, with an HA‐tag sequence added to the N‐terminus, was cloned into the pcDNA3.1 plasmid (Invitrogen) to construct the expression vectors. CHO cells were maintained in DMEM/F‐12 medium (Sigma‐Aldrich) supplemented with 10% fetal bovine serum (GE Healthcare) at 5% CO_2_ and 37°C. Before transfection, CHO cells (4 × 10^3^ cells/well) were detached and seeded into 96‐well plates. After 24 h, to assess the activity of either GUM3 or GUM4, cells were transiently transfected with 50 ng of the prepared expression vector, 200 ng of a firefly luciferase expression plasmid carrying the serum response element for the MAPK extracellular signal‐regulated kinase (ERK) signaling pathway (SRE‐Luc, Promega), and 50 ng of an expression plasmid for the *Renilla reniformis* luciferase reporter gene (pGL4.74, Promega) to determine transfection efficiency and serve as an internal control. All transfection procedures were carried out according to the manufacturer's instructions using Lipofectamine 2000 (Thermo Fisher Scientific) as the transfection reagent. After 24 h of transfection, cells were treated with appropriately diluted ligands to a final concentration of 1% DMSO and incubated at 37°C for 6 h. The cells were then rinsed with phosphate‐buffered saline. Promoter activity was quantified based on the luminescence of expressed luciferase, measured using a 2300 EnSpire plate reader (PerkinElmer) with the Dual‐Glo Luciferase Assay System (Promega).

### 4.5 Fluorescence Microscopy

Agonist‐dependent internalization of HA‐tagged GPR151 was observed by fluorescence microscopy using antibody fluorescence labeling. CHO cells were plated in 3.5 cm dishes (2.0 × 10^4^ cells/dish), cultured for 2 days, and then transiently transfected with the abovementioned expression vector to express GPR151. After 24 h of transfection, cells expressing GPR151 were stimulated with 100 μM ligand for 6 h. The cells were then fixed by incubation in 500 μL of 10% formalin for 20 min at room temperature and treated with 70% cold ethanol on ice for 1 h for permeabilization. After blocking with 1% bovine serum albumin in phosphate‐buffered saline, a primary antibody (anti‐HA‐tag (6B3) mouse immunoglobulin G monoclonal antibody, 3 μg/mL; IBL, Japan) was added and incubated at 4°C overnight. The following day, the primary antibody was removed, and cells were incubated with a fluorescent secondary antibody (fluorescein‐conjugated AffiniPure goat anti‐mouse immunoglobulin G, 5 mg/mL; Jackson ImmunoResearch, USA) at 4°C for 1 h. Finally, nuclei were stained using the NucBlue Fixed Cell ReadyProbes reagent (1/100 dilution; Thermo Fisher Scientific). Images were analyzed using a BZ‐9000 fluorescence microscope (Keyence, Japan) and processed with an analysis application to produce merged images of multiple stains.

### 4.6 Measurement of Antinociception in Rats

This experiment was approved by the Gunma University Animal Care and Experimentation Committee (Maebashi, Japan. No. 22‐063). All experiments were conducted in accordance with the guidelines of Animal Research and the study adhered to the ARRIVE guidelines ensuring compliance with ethical standards for animal research. Male Sprague–Dawley (SD) rats (300–450 g, SLC, Shizuoka, Japan) were used in the experiments. Animals were kept in a room with a temperature of 24°C ± 0.5°C, and a 12‐h cycle (8:00–20:00) of light and dark. Food and water were provided ad libitum. A total of 70 rats were used. Thermal nociceptive thresholds were measured using a plantar test apparatus (IITC; Life Science, USA) following methods similar to those previously reported (Hargreaves et al. 1988). Rats were placed in individual plastic boxes (10 × 20 × 24 cm) and allowed to acclimate for 30 min on the glass surface of the apparatus, which was maintained at 30°C. The PWL following application of an infrared radiant heat stimulus was measured using intense light focused on the hindlimb. The light intensity was adjusted to achieve a baseline latency of approximately 5 s for all animals. To prevent tissue damage during the analgesic period, the cutoff latency was set to 30 s. To eliminate the effects of significant individual differences, individuals with a latency of 15 s or more at 0 min postadministration were excluded from the analysis. According to predictions using ChemDraw (Version 19), the ClogP value for GUM3 was 3.914 and the logS value was −4.864, while the values for GUM4 were 4.727 and −5.661, respectively. These estimates suggest that these are hydrophobic compounds with low water solubility. Given the limited availability of these compounds and their low water solubility, the dose of GUM3 and GUM4 administered to rats was set at 1 mg/kg, which is effectively the upper limit of solubility. Before administration, the ligand compounds were prepared by dissolving them in DMSO, diluting with an equal volume of saline, and adding polyoxyethylene (20) sorbitan monooleate (Tween 80) to reach a final concentration of 0.2%. The PWL was measured three times at the plantar center of the right or left foot prior to drug administration (0 min) and at 30, 60, 90, and 120 min after drug administration. To eliminate individual differences in latency, the latency values were converted to %*MPE* using the following Equation (2):

(2) $$\%\mathrm{MPE}=\frac{\text{measured latency}-\text{baseline latency}}{\text{cutoff latency}-\text{baseline latency}}$$

All statistical analyses were carried out in Prism (GraphPad Prism 10, GraphPad Software Inc., San Diego, CA, USA). The data were analyzed using two‐way repeated‐measures ANOVA, followed by multiple comparisons. The *p*‐value was corrected using Bonferroni's method, and *p* < 0.05 was considered statistically significant. To measure the concentration of ligand compounds in the blood, a blood sample was collected every 30 min after drug administration, and serum was prepared. CSF was collected 30 and 120 min after administration. To avoid the effects of blood collection on behavior, separate rats were used for the antinociception test and blood collection. The concentration of each ligand compound in the serum was determined and quantified by tandem mass spectrometry (LC–MS/MS). The corresponding LC–MS/MS data were obtained using mass spectrometry (Xevo TQ Absolute, Waters, USA) coupled to an ultra‐high‐performance liquid chromatography system (ACQUITY UPLC, Waters) with a bridged ethyl hybrid C18 column (1.7 μm, 2.1 × 50 mm, Waters). Mobile phase A consisted of 0.1% formic acid in water, while mobile phase B consisted of 0.1% formic acid in acetonitrile. The gradient system was set as follows: 0 min at 2% B and 1.8 min at 98% B. The flow rate was 0.5 mL/min. Aliquots (25 μL) of serum samples obtained from each blood sample were treated with 50 μL of acetonitrile, and the resulting organic layer was injected into the LC–MS/MS system.

## Author Contributions

**Hijiri Yoshida:** data curation, formal analysis, investigation, visualization, validation. **Takashi Suto:** data curation, formal analysis, investigation, methodology, validation. **Hiroyuki Hirano:** validation, resources, software, writing – original draft. **Hiroyuki Osada:** formal analysis, methodology, resources, supervision, writing – review and editing. **Kazuto Nunomura:** data curation, formal analysis, methodology. **Bangzhong Lin:** data curation, investigation. **Shigeru Saito:** supervision, project administration. **Shigeki Takeda:** conceptualization, funding acquisition, project administration, validation, supervision, writing – original draft, writing – review and editing, visualization.

## Funding

This work was supported by Japan Society for the Promotion of Science (JSPS) KAKENHI (Grant Number JP25K08937) to S.T.

## Disclosure

All authors have reviewed the manuscript and agree to its submission.

## Ethics Statement

All animal experiments were conducted in compliance with the Gunma University Laboratory Animal Care Guidelines and were approved by the Animal Care and Use Committee of Gunma University (No. 22‐63).

## Conflicts of Interest

The authors declare no conflicts of interest.

## Supporting information


**Figure S1:** GPR151 ligand compounds identified in this study. NPD12440 (GUM3) and NPD13167 are first‐hit compounds selected from the RIKEN pilot library.
**Figure S2:** Structure–activity relationships inferred from activity measurements of 160 structural analogs of GUM3, as extracted from Table 3.
**Figure S3:** [^35^S]‐GTPγS binding assay resulted that the novel GPR151 ligands, GUM3 and GUM4, showed no ligand activity against the opioid receptors.
**Figure S4:** Even when co‐expressed with the opioid receptors and GPR151, the novel GPR151 ligands GUM3 and GUM4 did not alter the activity of the opioid receptors.

## Data Availability

The data that support the findings of this study are available on request from the corresponding author. The data are not publicly available due to privacy or ethical restrictions.
