# Supplementary material for: Novel Ligands for the Orphan Receptor GPR151 Modulate Morphine Action
Source: Genes Cells. 2026 May 8;31:e70119. doi: 10.1111/gtc.70119 (PMC13156533; doi:10.1111/gtc.70119)
Supplement: Supplementary file 1 — Figure S1: GPR151 ligand compounds identified in this study. NPD12440 (GUM3) and NPD13167 are first‐hit compounds selected from the RIKEN pilot library. Figure S2: Structure–activity relationships inferred from activity measurements of 160 structural analogs of GUM3, as extracted from Table 3. Figure S3: [35S]‐GTPγS binding assay resulted that the novel GPR151 ligands, GUM3 and GUM4, showed no ligand activity against the opioid receptors. Figure S4: Even when co‐expressed with the opioid receptors and GPR151, the novel GPR151 ligands GUM3 and GUM4 did not alter the activity of the opioid receptors. [file GTC-31-0-s001.pptx]

## Slide 1
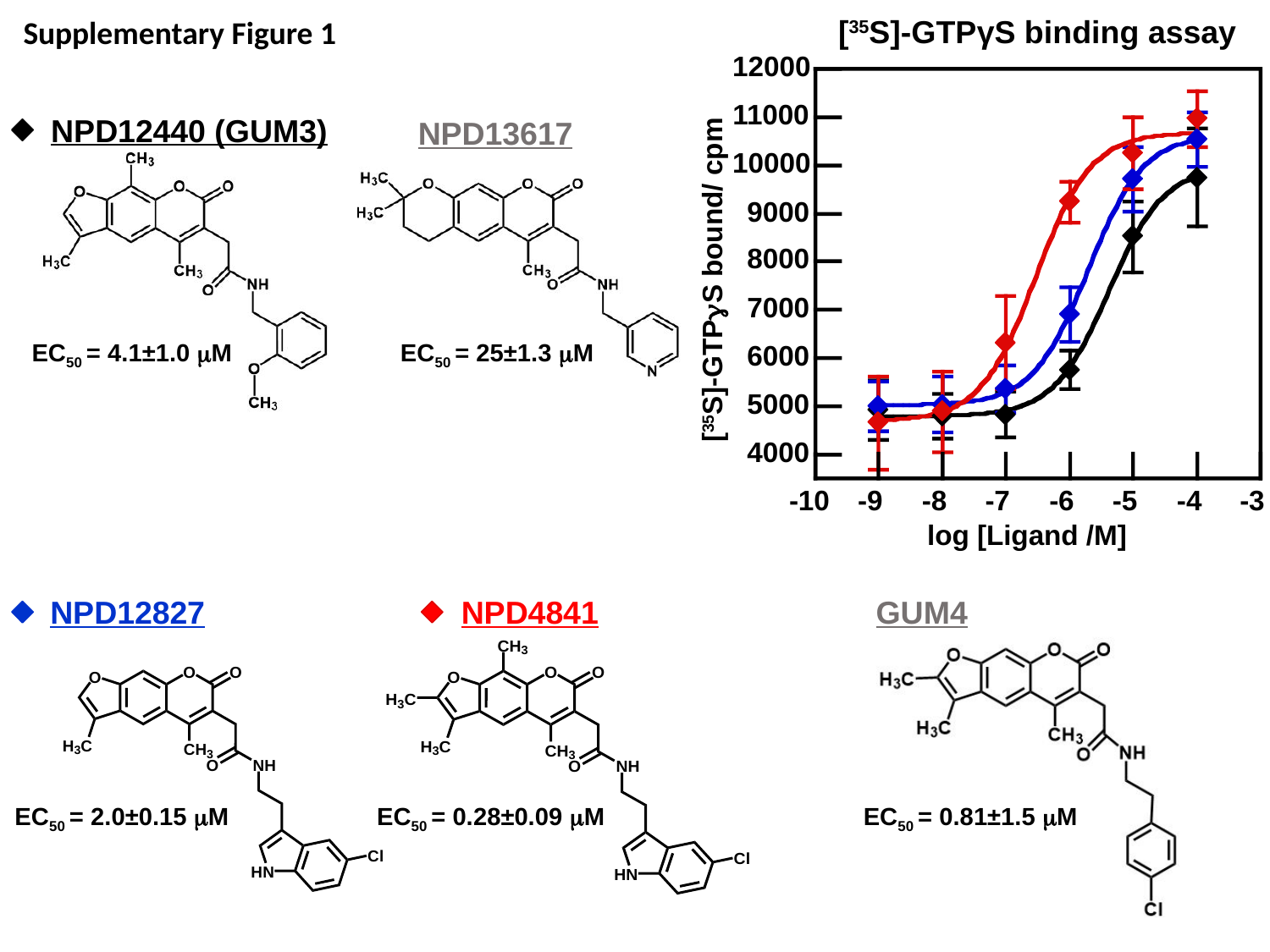

[35S]-GTPγS binding assay
Supplementary Figure 1
12000
11000
10000
9000
8000
[35S]-GTPgS bound/ cpm
7000
6000
5000
4000
-10
-9
-8
-7
-6
-5
-4
-3
log [Ligand /M]
NPD12440 (GUM3)
NPD13617
EC50 = 4.1±1.0 mM
EC50 = 25±1.3 mM
NPD12827
NPD4841
GUM4
EC50 = 2.0±0.15 mM
EC50 = 0.28±0.09 mM
EC50 = 0.81±1.5 mM

## Slide 2
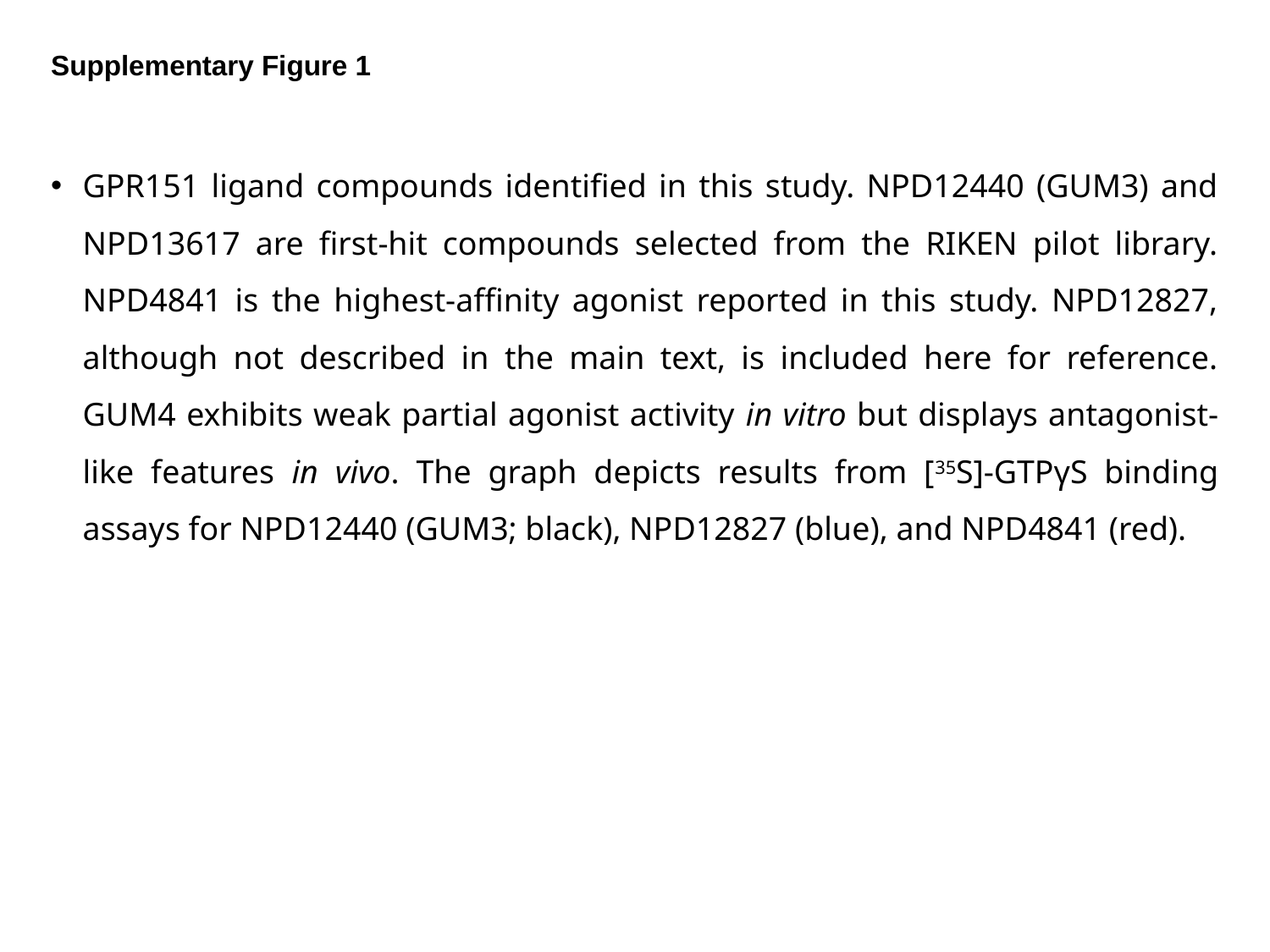

Supplementary Figure 1
GPR151 ligand compounds identified in this study. NPD12440 (GUM3) and NPD13617 are first-hit compounds selected from the RIKEN pilot library. NPD4841 is the highest-affinity agonist reported in this study. NPD12827, although not described in the main text, is included here for reference. GUM4 exhibits weak partial agonist activity in vitro but displays antagonist-like features in vivo. The graph depicts results from [35S]-GTPγS binding assays for NPD12440 (GUM3; black), NPD12827 (blue), and NPD4841 (red).

## Slide 3
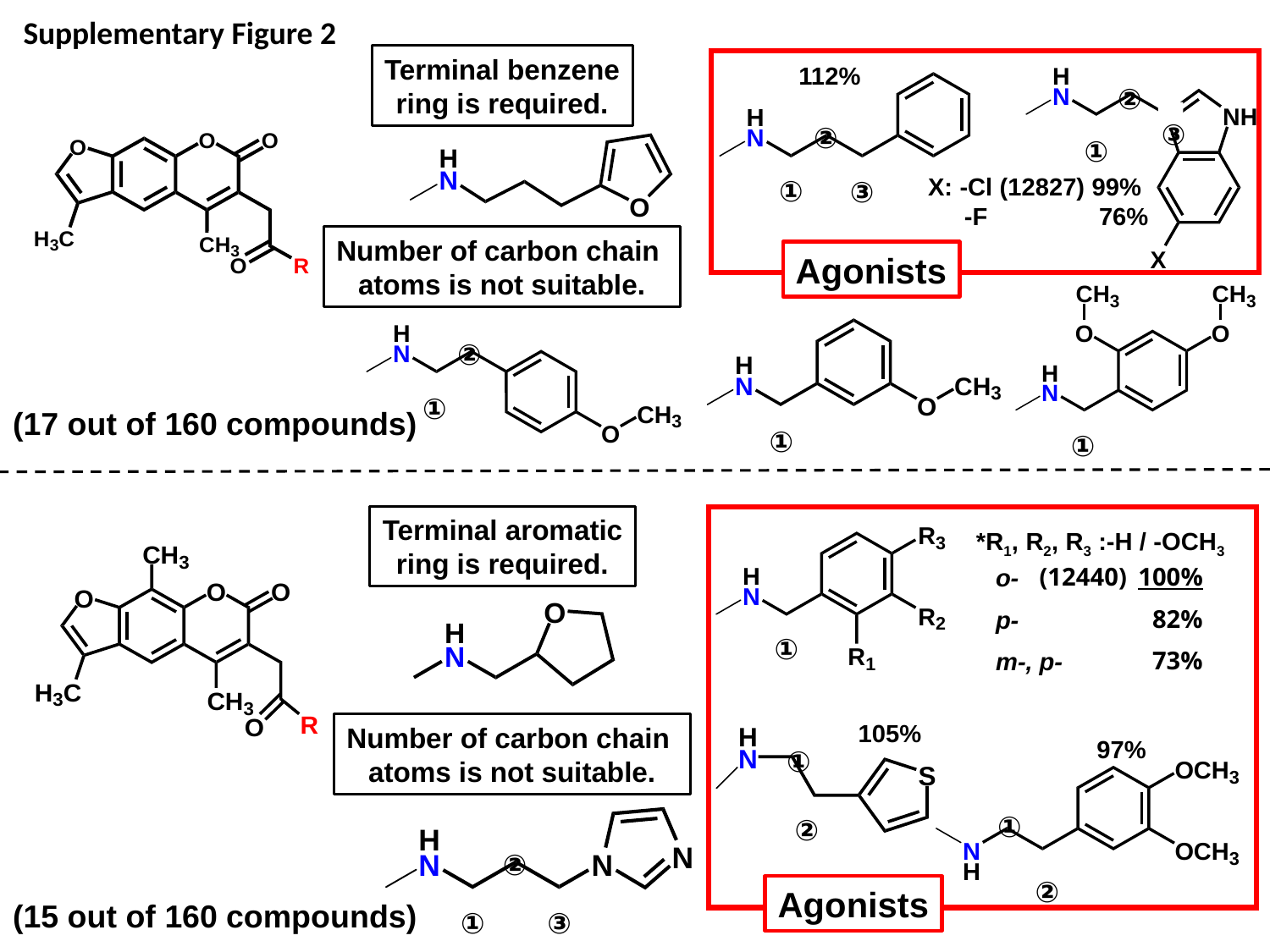

Supplementary Figure 2
Terminal benzene
ring is required.
112%
②
③
②
①
①
③
 X: -Cl (12827) 99%
　 -F 76%
Number of carbon chain
atoms is not suitable.
Agonists
②
①
(17 out of 160 compounds)
①
①
Terminal aromatic
ring is required.
*R1, R2, R3 :-H / -OCH3
 (12440)
100%
o-
82%
p-
73%
m-,
p-
①
105%
97%
①
①
②
②
Agonists
Number of carbon chain
atoms is not suitable.
②
①
③
(15 out of 160 compounds)

## Slide 4
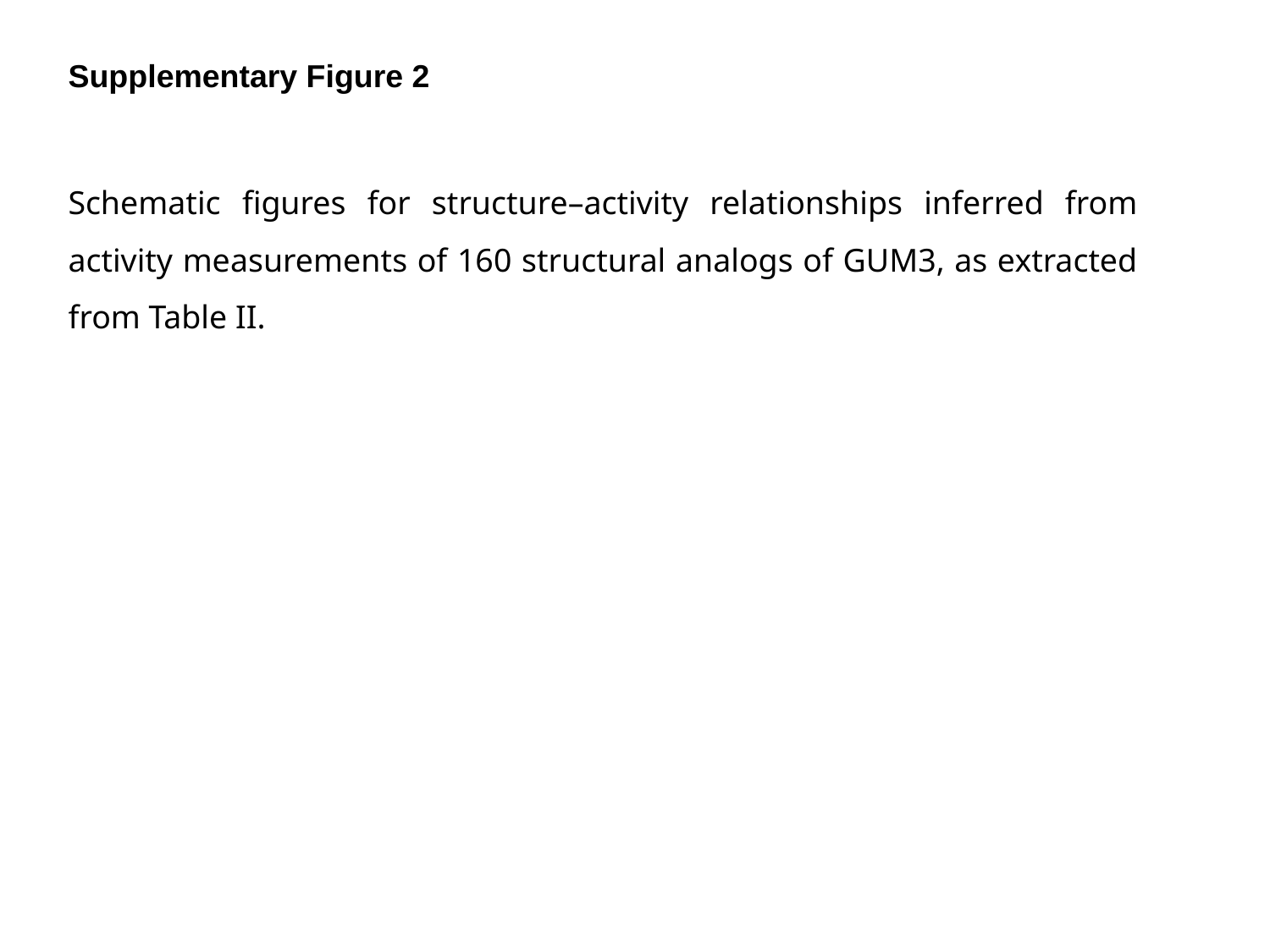

Supplementary Figure 2
Schematic figures for structure–activity relationships inferred from activity measurements of 160 structural analogs of GUM3, as extracted from Table II.

## Slide 5
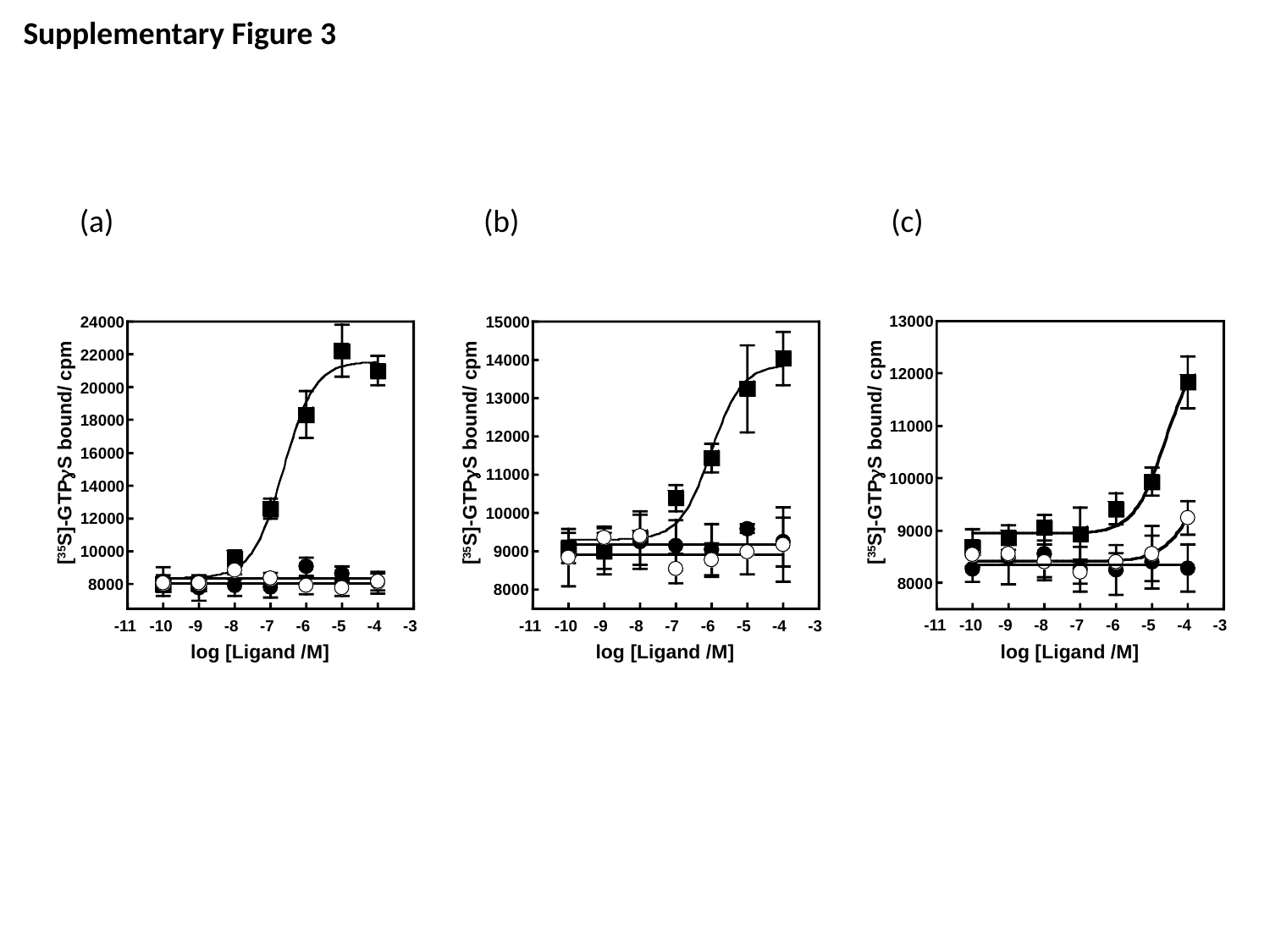

Supplementary Figure 3
(a)
(c)
(b)
13000
[35S]-GTPgS bound/ cpm
-11
-10
-9
-8
-7
-6
-5
-4
-3
log [Ligand /M]
12000
11000
10000
9000
8000
24000
22000
20000
18000
[35S]-GTPgS bound/ cpm
16000
14000
12000
10000
8000
-11
-10
-9
-8
-7
-6
-5
-4
-3
log [Ligand /M]
15000
14000
13000
12000
[35S]-GTPgS bound/ cpm
11000
10000
9000
8000
-11
-10
-9
-8
-7
-6
-5
-4
-3
log [Ligand /M]

## Slide 6
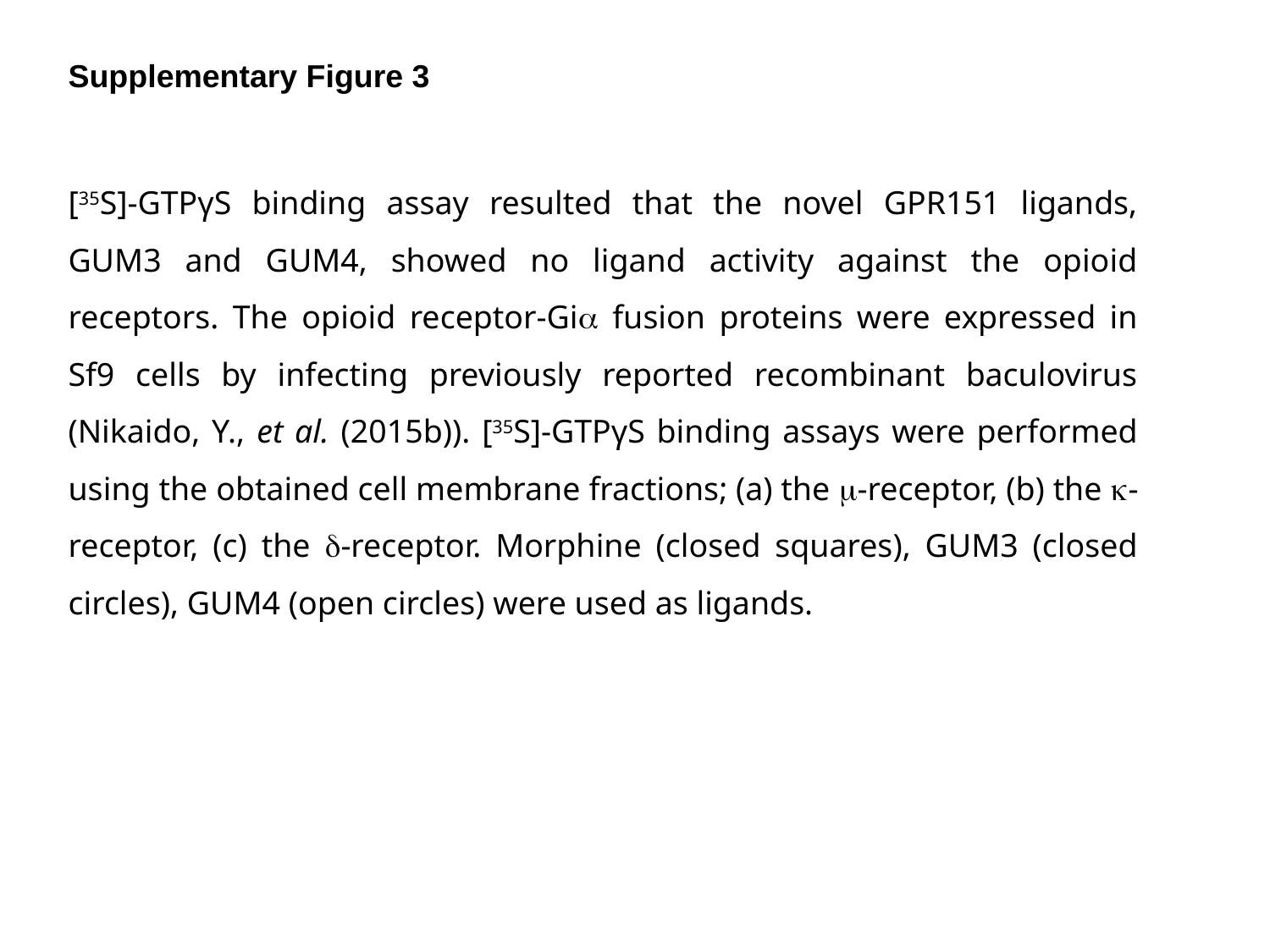

Supplementary Figure 3
[35S]-GTPγS binding assay resulted that the novel GPR151 ligands, GUM3 and GUM4, showed no ligand activity against the opioid receptors. The opioid receptor-Gia fusion proteins were expressed in Sf9 cells by infecting previously reported recombinant baculovirus (Nikaido, Y., et al. (2015b)). [35S]-GTPγS binding assays were performed using the obtained cell membrane fractions; (a) the m-receptor, (b) the k-receptor, (c) the d-receptor. Morphine (closed squares), GUM3 (closed circles), GUM4 (open circles) were used as ligands.

## Slide 7
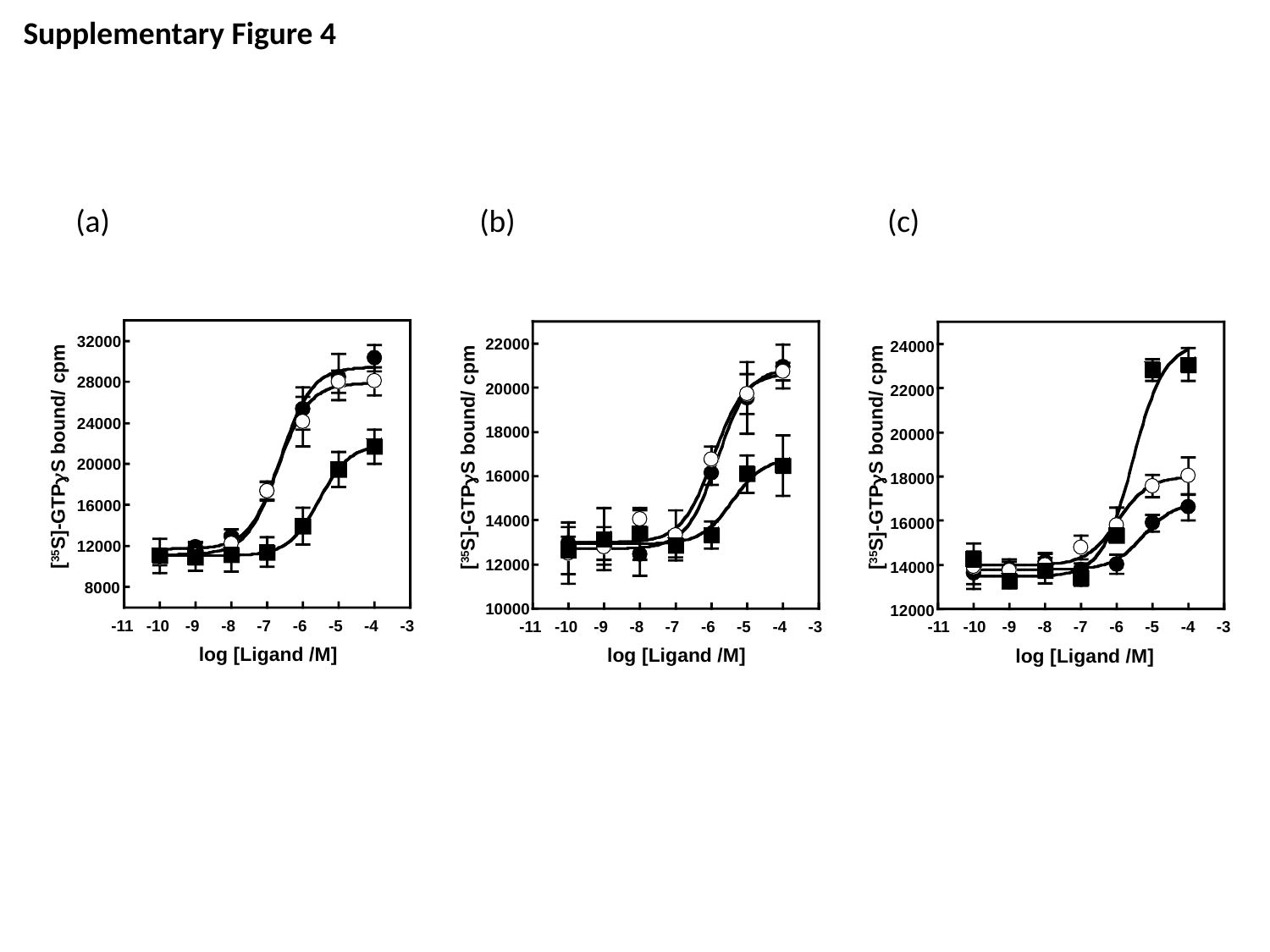

Supplementary Figure 4
(a)
(c)
(b)
32000
28000
24000
[35S]-GTPgS bound/ cpm
20000
16000
12000
8000
-11
-10
-9
-8
-7
-6
-5
-4
-3
log [Ligand /M]
22000
20000
18000
[35S]-GTPgS bound/ cpm
16000
14000
12000
10000
-11
-10
-9
-8
-7
-6
-5
-4
-3
log [Ligand /M]
[35S]-GTPgS bound/ cpm
-11
-10
-9
-8
-7
-6
-5
-4
-3
log [Ligand /M]
24000
22000
20000
18000
16000
14000
12000

## Slide 8
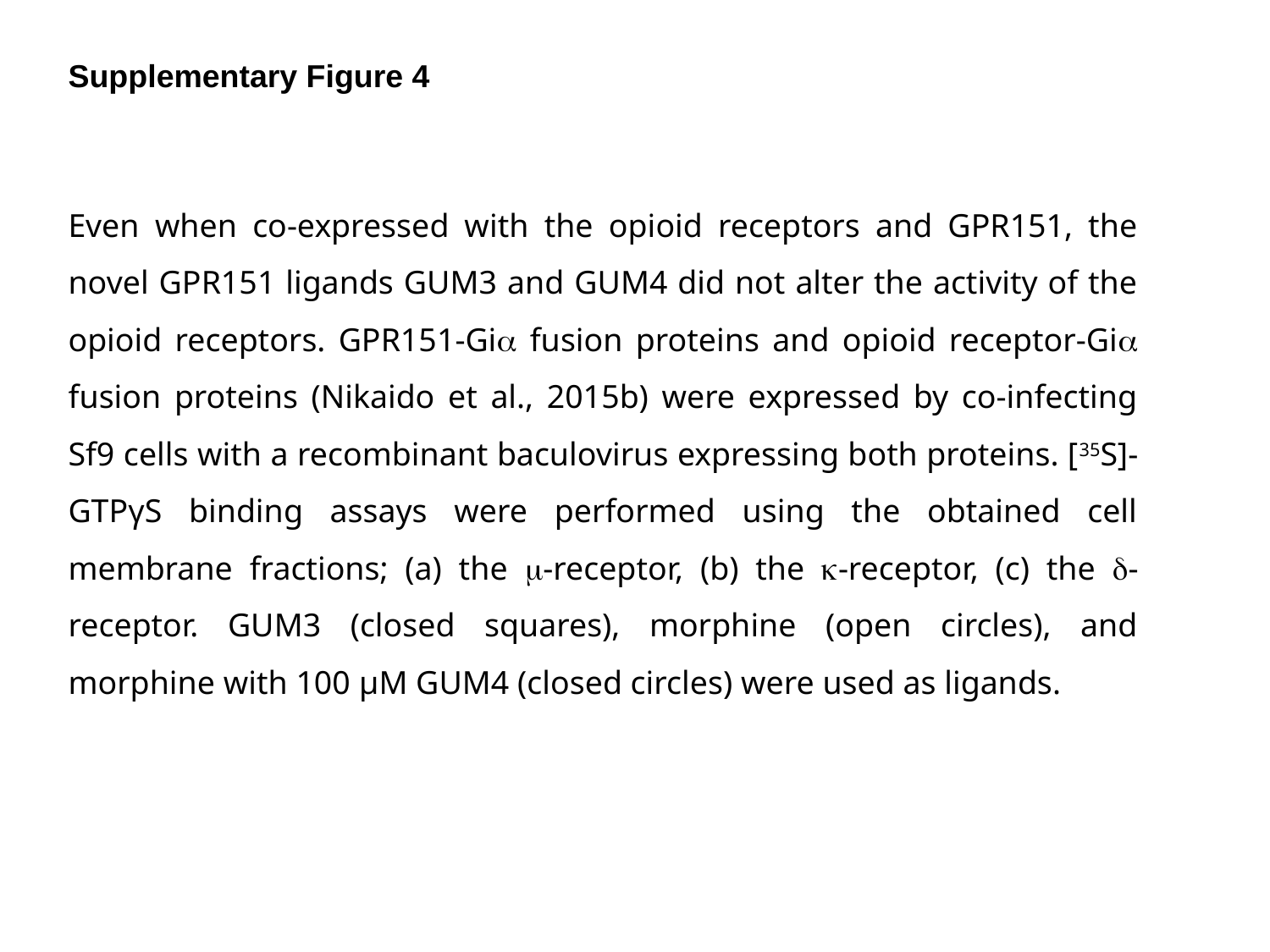

Supplementary Figure 4
Even when co-expressed with the opioid receptors and GPR151, the novel GPR151 ligands GUM3 and GUM4 did not alter the activity of the opioid receptors. GPR151-Giα fusion proteins and opioid receptor-Giα fusion proteins (Nikaido et al., 2015b) were expressed by co-infecting Sf9 cells with a recombinant baculovirus expressing both proteins. [35S]-GTPγS binding assays were performed using the obtained cell membrane fractions; (a) the -receptor, (b) the -receptor, (c) the -receptor. GUM3 (closed squares), morphine (open circles), and morphine with 100 μM GUM4 (closed circles) were used as ligands.
